# A Prescribing Cascade of Proton Pump Inhibitors Following Anticholinergic Medications in Older Adults With Dementia

**DOI:** 10.3389/fphar.2022.878092

**Published:** 2022-06-22

**Authors:** Shanna C. Trenaman, Austin Harding, Susan K. Bowles, Susan A. Kirkland, Melissa K. Andrew

**Affiliations:** ^1^ Department of Medicine, Dalhousie University, Halifax, NS, Canada; ^2^ Nova Scotia Health, Halifax, NS, Canada; ^3^ College of Pharmacy, Dalhousie University, Halifax, NS, Canada; ^4^ Department of Community Health and Epidemiology, Dalhousie University, Halifax, NS, Canada

**Keywords:** prescribing cascade, prescribing cascades, anticholinergic activity, proton pump inhibitor, dementia, prescribing quality, inappropriate medication, inappropriate medication prescriptions

## Abstract

**Introduction:** Prescribing cascade refers to use of a medication to treat a drug-related adverse event. Prescribing cascades increase medication use, cost, and risk of adverse events.

**Objective:** Our objective was to use administrative health data to identify whether use of medications from the anticholinergic cognitive burden scale was associated with proton pump inhibitor (PPI) prescribing consistent with a prescribing cascade in older adults with dementia.

**Method:** The cohort was comprised of Nova Scotia Seniors’ Pharmacare beneficiaries identified to have dementia and medication dispensation data recorded between 1 April 2010, or cohort entry and 31 March 2015. Anticholinergic medications from the anticholinergic cognitive burden scale (ACB) were abstracted. A look back period of 365 days identified if a PPI had been dispensed preceding anticholinergic dispensation. PPI initiation within 30, 60, 90, or 180 days of the anticholinergic medication was assessed. Demographic description of those dispensed anticholinergic medications or PPIs were reported. Risk factors for the prescribing cascade were investigated with logistic regression and Cox proportional hazards modelling including a sex-stratified analysis.

**Results:** We identified 28,952 Nova Scotia Seniors’ Pharmacare beneficiaries with dementia and prescription dispensation data. Anticholinergic medications were frequently dispensed with 63.4% of the cohort dispensed at least one prescription for an anticholinergic medication. The prescribing cascade defined as up to 180-days between anticholinergic medication inititation and PPI dispensation, occurred in 1,845 Nova Scotia Seniors’ Pharmacare beneficiaries with dementia (incidence 6.4%). Multivariate regression showed those experiencing the prescribing cascade after initiating any anticholinergic were younger (OR 0.98, 95%CI [0.97–0.98]), less likely to live in an urban location (OR 0.82, 95%CI [0.74–0.91]), or to be men (OR 0.74, 95%CI [0.67–0.82]). Cox regression demonstrated an increased risk of starting a PPI within 180 days when initiating any medication from the ACB (HR 1.38, 95%CI [1.29–1.58]).

**Discussion:** Regression modelling suggested that anticholinergic medications increased the risk of PPI dispensation consistent with a prescribing cascade in the cohort. The identification of the prescribing cascade in this population of older Nova Scotia Seniors’ Pharmacare Program beneficiaries with dementia using administrative health data highlights how routinely collected health data can be used to identify prescribing cascades.

## Introduction

The concept of the prescribing cascade was first reported by Rochon and Gurwitz in 1995 (Rochon and Gurwitz, 1995).

The prescribing cascade was defined as existing when an adverse drug event (ADE) was misinterpreted as a new medical condition that resulted in a new medication being prescribed to treat the ADE (Rochon and Gurwitz, 1995; Rochon and Gurwitz, 2017; McCarthy et al., 2019). Prescribing cascades can affect people of any age (Gill et al., 2005; Vouri et al., 2018; Huh et al., 2019; Vouri et al., 2020) but have been found to occur more frequently in older adults (Rochon and Gurwitz, 2017; McCarthy et al., 2019). This is due in part to increased polypharmacy among older compared to younger adults which increases exposure to drugs that potentially initiate the prescribing cascade (Beijer and de Blaey, 2002; Canadian Institute for Health Information, 2018). Older adults with dementia are even more susceptible to ADE than similarly aged controls without dementia as they often are prescribed an even greater number of medications (Kanagaratnam et al., 2017; Mullan et al., 2019). Prescribing cascades are an important public health issue. ADEs and inappropriate medication use can contribute to significant financial and health-related quality of life costs both of which affect health care systems and individuals (D’hulster et al., 2022; Mekonnen et al., 2021; Malakouti et al., 2021). Therefore, it is important from both clinical and policy perspectives to begin understanding how to prevent, detect, and reverse prescribing cascades (Brath et al., 2018).

As an example of a potentially relevant prescribing cascade which has yet to be thoroughly investigated, it has been proposed that older adults prescribed an increased anticholinergic burden were more likely to be prescribed a proton pump inhibitor (PPI) (Rababa et al., 2016). PPIs are the second most prescribed medication for older adults in Canada (Canadian Institute for Health Information, 2018) being used to treat a variety of stomach acid-related pathologies (Ahmed and Clarke, 2022). This high level of use raises concerns for overuse (Forgacs and Loganayagam, 2008; Farrell et al., 2017). In 2016, the Canadian Institute for Health Information reported that 23.6% of older adults using PPIs might have been using them inappropriately (Canadian Institute for Health Information, 2018). Concerns regarding overuse make PPIs a common target for deprescribing (the process of withdrawal of an inappropriate medication, supervised by a health care professional to manage polypharmacy and improve outcomes (Reeve et al., 2015)). PPI deprescribing is recommended in many cases after more than 8 weeks of therapy (Boghossian et al., 2017; Farrell et al., 2017; Williams et al., 2019; Deprescribing.org, 2022). Discontinuation of PPIs is recommended due to their association with increased risk of pneumonia (Lin et al., 2019; Marchina et al., 2019; Wang et al., 2019; Wongtrakul et al., 2020), deleterious effect on the gut microbiome (Minalyan et al., 2017; Kuo et al., 2021; Tsujimoto et al., 2021; Okuyama et al., 2022), poor outcome after COVID-19 infection (Yozgat et al., 2021; Ramachandran et al., 2022), fracture (Park et al., 2020; Veettil et al., 2022), *Clostridium difficile* infections (Kuo et al., 2021), and death (Brown et al., 2021; Aby et al., 2022).

Anticholinergic medication refers to a broad and diverse classification of medications (Nishtala et al., 2016; Villalba-Moreno et al., 2016) that includes, for example, antihistamines, antidepressants, and bladder anticholinergics. Anticholinergic medications antagonize the muscarinic receptors (subtypes 1 through 5) which are distributed throughout the body. Many medications have anticholinergic activity without the muscarinic receptor as the intended target receptor. The level of antagonistic activity varies between agents and can be measured using a variety of scales to quantify or rank the anticholinergic activity of medications with this property. There are many scales that quantify the anticholinergic activity of medications (Salahudeen et al., 2015; Al Rihani et al., 2021). The Anticholinergic Cognitive Burden (ACB) scale describes anticholinergic activity on a 4-point scale, with higher scores indicating stronger activity, and increased likelihood of ADE (Boustani et al., 2008). The ACB was chosen as it is a North American scale that was easily applied in the setting, offered a simple description of the anticholinergic activity as strong, moderate, or weak, and was freely available for use when the study was planned. Classical anticholinergic ADEs include dry mouth, decreased lower esophageal sphincter tone, urinary retention and constipation among others (Rudolph et al., 2008). More concerning for older adults is that anticholinergic medication exposure has been associated with an increased risk of falls (Ek et al., 2019; Shmuel et al., 2021), delirium (Oudewortel et al., 2021; Welk et al., 2022), dementia (Zheng et al., 2021) and poorer outcomes in those with dementia (Bishara et al., 2021; Oudewortel et al., 2021). This has led to recommendations for older adults to avoid anticholinergic medications but as this represents such a diverse group of medications it is challenging for prescribers to recognize these agents or even know which alternatives exist.

Rababa et al. proposed a novel prescribing cascade whereby anticholinergic induced gastrointestinal ADE were misinterpreted as new symptoms of gastroesophageal reflux and PPI prescription would follow. This was tested and identified in a cohort of older adults living in a long-term care home (Rababa et al., 2016), however it has not been more broadly investigated. Older adults living with dementia may have an impaired ability to explain their symptoms or perhaps recount with clarity when gastrointestinal symptoms begin, making it exceedingly challenging for clinicians to recognize the potential prescribing cascade of anticholinergic induced gastrointestinal ADE thereupon being treated with a PPI. The prescribing cascade of anticholinergic medication exposures in older adults with dementia leading to PPI prescription is the focus of the present study. The hypothesis to be explored is that there is increased prescribing of PPIs temporally associated with initiation of a strongly anticholinergic medication. Our objective was to determine if there is an association between anticholinergic medication initiation and PPI prescribing consistent with a prescribing cascade in older adults with dementia.

## Materials and Methods

### Data Description

Health Data Nova Scotia (HDNS) provided linked administrative claims data extracted from provincial data sources including Medical Services Insurance Physician’s Billings (MED), Seniors’ Pharmacare (PHARM), Vital Statistics (VITAL), and the Canadian Institute for Health Information—Discharge Abstract Database (DAD). The MED provided details of medically required hospital visits for medical, dental, and optometric services with some restrictions for eligible residents. The DAD captured administrative, clinical, and demographic information on hospital discharges in Canada. The PHARM database catalogued dispensing data for Nova Scotia Seniors’ Pharmacare beneficiaries. Nova Scotia Seniors’ Pharmacare is a voluntary provincial drug insurance program that covers a formulary of prescription medications and is available to adults over 65 years of age in Nova Scotia. VITAL database provided the date of death for censoring.

**FIGURE 1:**
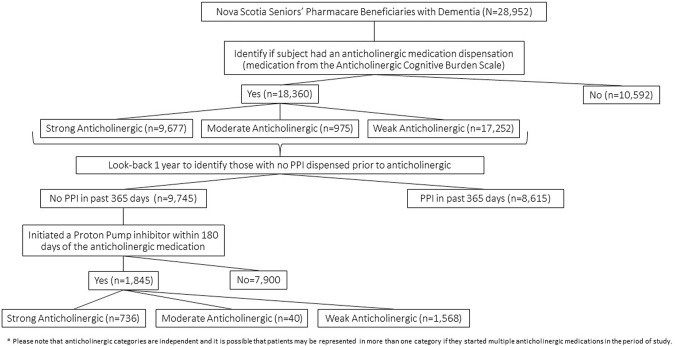
Patient flow through study analytic procedure.

Cohort entry was assigned when an eligible Nova Scotia Seniors’ Pharmacare beneficiary was identified to have had any one of the International Classification of Diseases Clinical Modification (ICD) 9/10 codes that identify dementia from the MED or DAD databases within the date range of 1 March 2005, to 31 March 2015, to create the most complete cohort of older adults with dementia in the province as possible. The particular ICD 9/10 codes used to define dementia were previously identified by the Nova Scotia Dementia Strategy (Dementia Strategy, 2021) (Supplementary Table S1). At cohort entry, data collection included the sex of the subject, first date of dementia diagnosis identified in the observation period, and the geographic location of residence specified by the second digit of the postal code whereby 0 represents a rural location and digits 1–9 represent urban sites (Nova Scotia, 2022). Once meeting cohort entry criteria, prescription drug dispensation data for anticholinergic medications according to the ACB scale (Boustani et al., 2008) was collected over the five-year period from 1 April 2010, to 31 March 2015. The PHARM database provided PPI dispensation data from cohort entry or 1 April 2009, to 31 March 2015, which allowed a look-back period of 1 year to test that PPI dispensation followed the anticholinergic medications. A 1-year look-back period was considered adequate to allow for the a new PPI prescription to be related to a new indication and the likelihood that a PPI was needed again due to an underlying medical condition would be similar in both those initiating anticholinergics and those not on an anticholinergic. Exposure to a medication was defined as any dispensation according to the PHARM record, with the required assumption that dispensation was equivalent to medication use. The PHARM data included medication name, quantity dispensed, days supplied, and prescription fill date. Cohort exit was at the date of death or study end date of 31 March 2015. Figure 1 shows the flow of patient subjects through the analytic procedures for reference.

### Analytic Procedure

From 1 April 2010, or cohort entry which could occur up until 31 March 2015, details of medication dispensation for anticholinergic medications were abstracted from the PHARM database, including details of the strength of the anticholinergic according to the ACB scale (Boustani et al., 2008). A look-back period of 365 days from the first date of dispensation of an anticholinergic medication from the ACB scale was used to identify if a PPI had been dispensed in the year preceding the first recorded anticholinergic dispensation. Once confirmed that a PPI did not precede the anticholinergic medication, a forward look in time appraised for PPI initiation within 30, 60, 90, or 180 days of the anticholinergic medication. A stratified analysis was then repeated, categorizing by strength (strong, moderate, and weak) of anticholinergic medications according to the ACB scale. Patient characteristics of those experiencing the prescribing cascade were explored using descriptive statistics. Logistic regression (crude and adjusted) was used to identify risk factors for the prescribing cascade (sex, age at dementia diagnosis, rural or urban location of residence). We then used a Cox proportional hazards model to explore being dispensed a PPI as the outcome of interest in a survival analysis. This method allowed comparison of those who were prescribed a PPI and those who did and did not receive an anticholinergic medication prior to PPI initiation. Time to event was considered from the date of the anticholinergic medication prescription to the dispensing of the PPI, with comparisons made for those dispensed and not dispensed an anticholinergic medication with censoring at 180 days. Missing data were handled using case-wise deletion.

### Statistical Software

All data analyses were completed on STATA version 15.1, StataCorp, Lakeway Drive, College Station, Texas, United States.

## Results

In the period from 1 April 2005, to 31 March 2015, there were 28,952 (17,946 women (62.0%) and 10,528 men (36.4%)) Nova Scotia Seniors’ Pharmacare beneficiaries identified to have a dementia diagnosis. The average age at dementia diagnosis was 81.1 years (95% CI: 81.0–81.2) with women being slightly older than men [mean 82.1 years (95% CI: 82.0–82.2) compared to 79.6 years (95% CI: 79.4–79.7) (*p* < 0.00001)]. At cohort entry 32.3% of the cohort resided in a rural location. The mean duration of follow-up in the cohort was 3.6 years.

**FIGURE 2:**
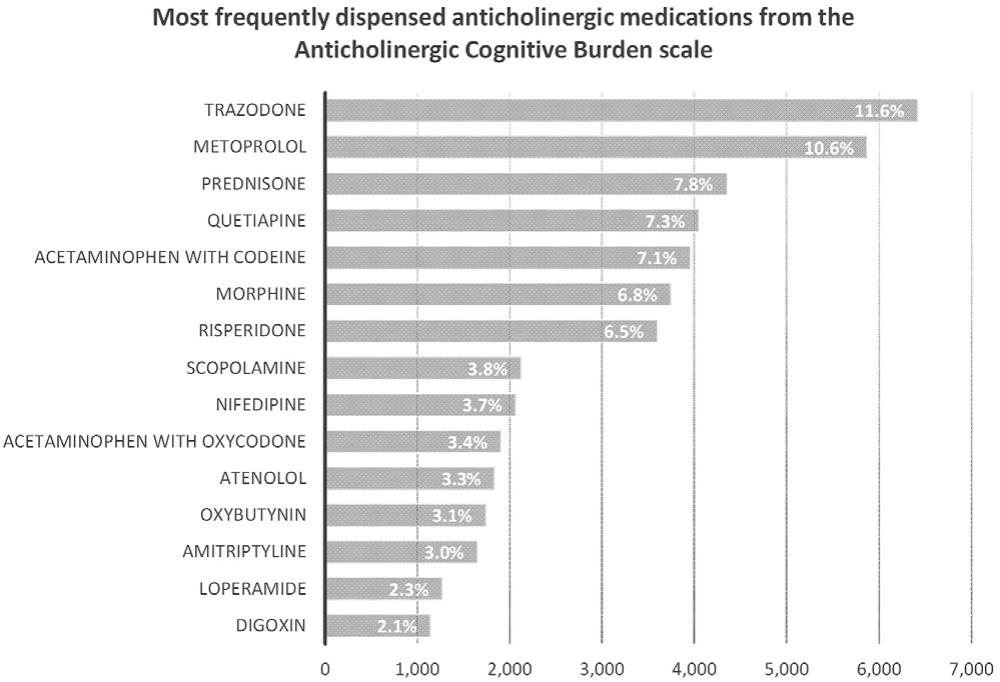
Number and percent of Nova Scotia Senior’s Pharmacare Beneficiaries with dementia dispensed at least one medication from the Anticholinergic Cognitive Burden scale for the top fifyeen most frequently dispensed medications.

We describe the number of Nova Scotia Seniors’ Pharmacare Beneficiaries with dementia dispensed at least one anticholinergic medication or PPI between 1 April 2010, and 31 March 2015, in Table 1. The most frequently dispensed anticholinergic medications are summarized in Figure 2; a detailed list of the anticholinergic medications dispensed is presented in Appendix Table A1. More than 75% of those dispensed any anticholinergic medication were dispensed more than one over the period of study. The average number of anticholinergic medications dispensed to those receiving least one medication from the ACB scale was 3 (range 1–14). PPIs dispensed included: rabeprazole (n = 4,539, 50.4%), omeprazole (n = 2,552, 27.8%), pantoprazole (n = 1,823, 20.0%), lansoprazole (n = 148, 1.7%) and esomeprazole (n = 10, 0.1%).

**TABLE 1:** Anticholinergic and Proton Pump Inhibitor (PPI) Dispensation including Prescribing Cascade Occurrence in the cohort of older adults with dementia.

Subjects (n = 28,952)	Any anticholinergic	Anticholinergic level 3 (strong)	Anticholinergic level 2 (Moderate)	Anticholinergic level 1 (weak)	PPI
Number of subjects with at least one dispensation, n	18,360	9,677	975	17,252	10,559
Age at diagnosis mean (standard deviation)	81.1 (7.9)	80.6 (7.9)	78.6 (8.3)	81.2 (7.9)	80.9 (7.9)
Female sex n (%)	12,411 (68.5)	6,760 (70.7)	614 (63.9)	11,670 (68.5)	7,078 (68.1)
Rural location of residence, n (%)	6,407 (34.9)	3,433 (35.5)	340 (34.9)	5,990 (34.7)	3,789 (35.9)
Prescribing cascade PPI prescribed within 180 days	1,845	736	40	1,568	—
Women n (%)	1,230 (66.7%)	523 (71.0%)	19 (47.5%)	1,027 (65.5%)	—
Prescribing cascade within 90 days	1,417	544	26	1,178	—
Women n (%)	969 (68.4%)	397 (73.0%)	11 (42.3%)	788 (66.9%)	—
Prescribing cascade within 60 days	1,174	457	22	958	—
Women n (%)	810 (69.0%)	339 (74.1%)	8 (36.4%)	644 (67.2%)	—
Prescribing cascade within 30 days	780	306	15	637	—
Women n (%)	549 (70.4%)	232 (75.8%)	<5	440 (69.1)	—

We identified 1,845 cases where older adults with dementia initiated a PPI within 6 months (180 days) of starting an anticholinergic medication (Table 1). Examining the strength of the anticholinergic medications according to the ACB scale shows that most medications were in the strong and weak categories with dispensations to women predominating in both of those categories. However, men who met the criteria for the prescribing cascade were more commonly dispensed medications in the moderate activity category prior to PPI initiation. An exploration of the robustness of the prescribing cascade association by reducing the interval from 180 to 90, 60, and 30 days is displayed in Table 1. More than 50% of the identified cases of the prescribing cascade occurred within 60 days of the anticholinergic medication prescription being dispensed.

Multivariate regression (Table 2) showed those experiencing the prescribing cascade after initiating any anticholinergic (initiated a PPI 1–180 days of an anticholinergic medication) were younger (OR 0.98, 95%CI: 0.97–0.98), less likely to live in an urban location (OR 0.82, 95%CI: 0.74–0.91), and less likely to be men (OR 0.74, 95%CI: 0.67–0.82). Crude estimates were similar for age (OR 0.98, 95%CI: 0.97–0.98), rurality (OR 0.77, 95%CI: 0.70–0.85), and the effect of sex (OR 0.79, 95%CI: 0.71–0.87). Analyses limited to strong anticholinergic medications showed similar trends with age, with younger adults (OR 0.96, 95%CI: 0.95–0.97) and those living in a rural location (OR 0.86, 95%CI: 0.73–0.99) being more likely to experience a prescribing cascade, although the association with sex was not maintained (OR 0.58, 95%CI: 0.3–1.46). Analyses limited to moderate anticholinergic medications showed similar trends with age (OR 0.91, 95%CI: 0.87–0.95), whereas rurality (OR 1.04, 95%CI: 0.53–2.02) and sex (OR 1.47, 95%CI: 0.79–44.8) failed to show statistically significant associations. Use of weak anticholinergic medications showed similar trends as overall with younger age (OR 0.98, 95%CI: 0.97–0.98), those living in a rural location (OR 0.83, 95%CI: 0.75–0.92), and women (OR 0.79, 95%CI: 0.71–0.88) being associated with increased odds of meeting the criteria of the prescribing cascade.

**TABLE 2:** Multivariate regression findings for relationships between the prescribing cascade and potential risk factors.

Covariates	Unadjusted odds ratio (95% CI)	Adjusted odds ratio (95%CI)
Any Anticholinergic		
Age	0.98 (0.97–0.98)	0.98 (0.97–0.98)
Urban	0.77 (0.70–0.85)	0.82 (0.74–0.91)
Male Sex	0.79 (0.71–0.87)	0.74 (0.67–0.82)
Strong Anticholinergic		
Age	0.96 (0.95–0.97)	0.96 (0.95–0.97)
Urban	0.80 (0.69–0.93)	0.86 (0.73–0.99)
Male Sex	0.64 (0.54–0.75)	0.58 (0.30–1.46)
Moderate Anticholinergic		
Age	0.91 (0.87–0.94)	0.91 (0.87–0.95)
Urban	0.93 (0.48–1.81)	1.04 (0.53–2.02)
Male Sex	1.85 (0.99–3.44)	1.47 (0.79–44.8)
Weak Anticholinergic		
Age	0.98 (0.97–0.99)	0.98 (0.97–0.98)
Urban	0.78 (0.70–0.86)	0.83 (0.75–0.92)
Male Sex	0.84 (0.75–0.93)	0.79 (0.71–0.88)

Cox regression (Table 3) demonstrated an increased risk of starting a PPI within 180 days of initiating an anticholinergic from the ACB scale (HR 1.38, 95%CI: 1.29–1.58), and an even greater risk for those dispensed a strong anticholinergic (HR 6.57, 95%CI: 5.45–7.97), but not a moderate anticholinergic (HR 1.63, 95%CI: 0.68–3.88). There was an increased risk of the prescribing cascade for those dispensed a weak anticholinergic (HR 1.38, 95%CI: 1.25–1.82) but much less of an association than identified for the stronger agents, consistent with the hypothesis. In a sex-stratified analyses, the prescribing cascade was identified to exist in men with a significantly increased risk for PPI after anticholinergic medication (HR 1.27 95%CI: 1.06–1.53) and even more so for women (HR 1.43 95%CI: 1.29–1.66).

**TABLE 3:** Cox regression results for likelihood of initiating a PPI within 180 days of an anticholinergic medication from the Anticholinergic Cognitive Burden Scale.

Anticholinergic medication category	Unadjusted hazard ratio (95% CI)
Any Anticholinergic	1.38 (1.29–1.58)
Strong Anticholinergic	6.57 (5.45–7.97)
Moderate Anticholinergic	1.63 (0.68–3.88)
Weak Anticholinergic	1.38 (1.25–1.82)

## Discussion

We found evidence for a prescribing cascade of anticholinergic medications leading to PPI prescription in this cohort of older adults living with dementia. Both anticholinergic medications and PPIs were frequently dispensed; PPIs were dispensed to more than 25% of the cohort. Weak anticholinergic medications and strong anticholinergic medications were the most frequently dispensed. Overall, 63.4% of the cohort were dispensed at least one prescription for an anticholinergic medication despite these being potentially harmful for this vulnerable population. We found 1,845 instances of the prescribing cascade, as defined by up to a 180-days interval between anticholinergic medication and PPI dispensation among the 28,952 older adults with dementia included in the cohort (incidence 6.4%). The logistic regression and stratified Cox regression results suggest that this prescribing cascade was most common in older women with dementia.

PPIs are commonly prescribed and their use has increased since 2014 with monthly prescription prevalence estimated at 11,000 per 100,000 persons in the United Kingdom Clinical Practice Research Datalink in 2018 (Abrahami et al., 2021). This level of PPI use from the United Kingdom Clinical Practice Research Datalink is much lower than the rate of use in our cohort of older adults with dementia, which we estimate to be at 40,223 per 100,000 persons in the final month of analysis (March 2015 had 3,711 PPI prescriptions written to the cohort sized at 9,226 in that month of observation). This may reflect that our selected population is older, living with more comorbidities, and therefore more likely to be prescribed gastro-protection with a PPI.

Anticholinergic medications from the ACB scale were dispensed to 63.4% of the cohort, reflecting high levels of use. This is concerning given the known risks of anticholinergic medication exposure to older adults with dementia in worsening cognitive outcomes, causing delirium, falls and increasing the risk of dementia (Bishara et al., 2021). Weak anticholinergic medications were the most commonly dispensed, most likely as this group includes many common medications for managing chronic conditions (e.g., metoprolol and warfarin) and reflects medication choices that may be harder to discontinue, switch to other agents and potentially will be lowest risk of cause ADE. Quite concerning are high rates of trazodone, quetiapine, risperidone, and amitriptyline dispensation (Supplementary Table S1). These medications are highly anticholinergic, deliriogenic and likely to have an unfavourable risk benefit profile for older adults with dementia. These represent four medications that within our jurisdiction were frequently prescribed and rather than targeting PPI dispensation suggests that actually targeting the frequent prescribing of anticholinergic medications is of a greater importance for improved prescribing.

The identification of the anticholinergic-PPI prescribing cascade in this population of older Nova Scotia Seniors’ Pharmacare Program beneficiaries with dementia using administrative health data highlights how routinely collected health data can be used to identify and investigate prescribing cascades. It is important to consider that the prescribing cascade as related to a medication with anticholinergic activity precipitating prescription of a PPI may not always be inappropriate. It is possible that the prescribing cascade identified may reflect an appropriate prescribing decision such as initiating a PPI as gastroprotection after initiating a selective serotonin reuptake inhibitor or prednisone. Even with the possibility of some prescribing cascades being appropriate the methodology used in this study identifies a mechanism by which theoretical prescribing cascades could be examined using administrative health data. Once identified the information could be used to target a reduction in inappropriate prescribing after communication to prescribers or to develop interventions to address possible inappropriate prescribing.

How to address this prescribing cascade or others like it and reduce its risk of occurrence will also take effort from prescribers and collaboration from all members of the healthcare team. An Ontario-based qualitative study investigated the patient and provider perspectives on prescribing cascades in community-dwelling adults aged 65 and older (Farrell et al., 2020). Using semi-structured interviews with patients, pharmacists, and physicians evolving themes were identified in consideration of best ways to resolve prescribing cascades. The three main themes were lack of awareness of the prescribing cascade, uncertainty regarding provider/patient accountability, and lack of available information or ability to collaborate. In recognizing these themes, the authors indicated nine actions some of which include patient empowerment, increasing the role for pharmacists to facilitate prescribing and monitoring, using alerts in prescribing and dispensing software, and incorporation of current prescribing pitfalls and prescribing cascades into medical education. These actions can further be condensed to prevention, detection, and reversal. The implementation of these strategies will be better executed with a cohesive, collaborative strategy supported by health structures including healthcare providers and regular assessment of administrative health data.

A commonly asked question is how harmful prescribing cascades can be reversed. Exploring the themes identified by Farrell et al. a scoping review focusing on the prevention, detection, and reversal of prescribing cascades (Brath et al., 2018) showed that successful strategies for prevention include patient education and empowerment and providing providers with a list of cascades with additional guidance to start with low doses of medications when prescribing cascade implicated medications must be used. In general, principles that support deprescribing also support detection and avoidance of prescribing cascades. Primary care practices have found success in identifying potentially inappropriate medication use when a pharmacist was integrated into interprofessional care teams. For example, a study from Quebec, Canada assessed the impact of pharmacists integrated into Family Medicine Groups. Within the Family Medicine Groups pharmacists performed medication reviews that detected 300 drug related problems (an average of 7.2 per patient), with the most common being ‘drug use without indication’ (27%) (Khaira et al., 2020). Unfortunately to date, there is not a robust healthcare system-entrenched method for supporting patients in deprescribing. Pharmacists have the skills to support deprescribing but may lack access to essential personal health information and an effective means of providing collaborative and coordinated deprescribing services.

### Limitations

Our study is not without limitations. As our analyses were based on administrative data, we lacked the ability to assess clinical factors or indications that entered the prescribing decisions. Additionally, we relied on dispensation data which does not provide details as to whether medications were taken as prescribed, or if taken how successfully prescription directions were adhered to. We did not have access to details of over-the-counter medications some of which are anticholinergic (e.g., antihistamines and muscle relaxers). We also do not know if non-prescription PPIs were self-selected and used rather than obtaining a prescription PPI. Our population of Nova Scotia Seniors’ Pharmacare beneficiaries covers about 63% of adults 65 years of age and older in Nova Scotia. Nova Scotia Seniors’ Pharmacare beneficiaries include those who have enrolled in the voluntary insurance program and is less likely to include those with private insurance that continues after retirement and those who do not wish to pay for medication insurance due to perception of low need, low income, or low literacy.

## Conclusion

The use of highly anticholinergic medications in older adults who live with dementia is a concern due to the adverse events associated with their use. Identification of the prescribing cascade associated with anticholinergic medications leading to PPI prescription in older adults living with dementia is only one component of the potential solution. Avoiding prescription of potently anticholinergic medications or having pharmacists act on alerts when strongly anticholinergic medications are prescribed is likely to be most successful for reducing complications associated with anticholinergic medication use like the prescribing cascade described. If anticholinergic medications are prescribed avoiding a prescribing cascade will require empowerment of patients or care providers by providing them with the tools and education to identify adverse events, consistent messaging and follow up to evaluate tolerance and potential ADE when new medications are started. Success in prescribing cascade management will likely only be achieved when methods for interdisciplinary communication and interventions are created and supported by health data evaluation and structured cross-discipline communication. Some of these ideals may be realized when we determine how to share e-health records among providers to allow for seamless transfer of care between providers, expansion of drug utilization evaluation and routine assessment of prescribing indicators to capture prescribing trends with increased use of prescribing alerts to warn when a potential prescribing cascade is identified. Ideally, legislation or practice agreements would support these measures and provide a framework for collaboration to the outcome of improved prescribing.

## Data Availability

The raw data will be made available upon reasonable request once approved by the Research Ethics board and data custodian HDNS.

## Appendix 1

**TABLE A1:** Anticholinergic Cognitive Burden Scale Medications dispensed to the cohort of Nova Scotia Seniors’ Pharmacare Beneficiaries with Dementia.

Anticholinergic medication		Frequency	% of anticholinergic claims
ATC code	Generic name		
Anticholinergics_3			
A03AA07	dicycloverine	50	0.09
A04AD01	scopolamine	2,120	3.82
A04AD99	dimenhydrinate	415	0.75
G04BD04	oxybutynin	1,740	3.13
G04BD07	tolterodine	377	0.68
G04BD08	solifenacin	204	0.37
G04BD09	trospium	57	0.10
G04BD10	darifenacin	34	0.06
G04BD11	fesoterodine	31	0.06
M03BC01	orphenadrine (citrate)	6	0.01
N04AA01	trihexyphenidyl	28	0.05
N04AA04	procyclidine	6	0.01
N04AC01	benzatropine	162	0.29
N05AB03	perphenazine	92	0.17
N05AB06	trifluoperazine	52	0.09
N05AH02	clozapine	5	0.01
N05AH03	olanzapine	480	0.86
N05AH04	quetiapine	4,047	7.29
N05BB01	hydroxyzine	152	0.27
N06AA01	desipramine	65	0.27
N06AA02	imipramine	80	0.12
N06AA04	clomipramine	50	0.14
N06AA06	trimipramine	38	0.07
N06AA09	amitriptyline	1,650	2.97
N06AA10	nortriptyline	671	1.21
N06AA12	doxepin	254	0.46
N06AB05	paroxetine	1,053	1.90
Anticholinergics_2			
N02AB02	pethidine	10	0.02
N03AF01	carbamazepine	328	0.59
N04BB01	amantadine	67	0.12
N05AA02	levomepromazine	468	0.84
N05AG02	pimozide	15	0.03
N05AH01	loxapine	123	0.22
Anticholinergics_1			
A02BA01	cimetidine	52	0.09
A07DA03	loperamide	1,271	2.29
B01AC07	dipyridamole	16	0.03
C01AA05	digoxin	1,141	2.06
C01DA08	isosorbide dinitrate	110	0.20
C01DA14	isosorbide mononitrate	274	0.49
C03BA04	chlortalidone	34	0.06
C07AB02	metoprolol	5,869	10.57
C07AB03	atenolol	1,836	3.31
C07CB03	atenolol and other diuretics	76	0.14
C08CA05	Nifedipine	2,063	3.72
C09AA01	captopril	39	0.07
H02AB07	prednisone	4,350	7.84
M04AC01	colchicine	997	1.80
N01AH01	fentanyl	15	0.03
N02AA01	morphine	3,745	6.75
N02AA59	codeine, combinations excl. psycholeptics	3,955	7.12
N02AB03	fentanyl	359	0.65
N02BE51	paracetamol, combinations excl. psycholeptics	1,905	3.43
N05AX08	risperidone	3,598	6.48
N05BA01	diazepam	493	0.89
N05BA05	potassium clorazepate	21	0.04
N05BA12	alprazolam	405	0.73
N06AX05	trazodone	6,418	11.56
N06AX12	bupropion	425	0.77
R03DA04	theophylline	129	0.23
R03DA54	theophylline, combinations excl. psycholeptics	13	0.02
R05DA04	codeine	974	1.75
