# Supplementary material for: A Prescribing Cascade of Proton Pump Inhibitors Following Anticholinergic Medications in Older Adults With Dementia
Source: Front Pharmacol. 2022 Jun 22;13:878092. doi: 10.3389/fphar.2022.878092 (PMC9257131; doi:10.3389/fphar.2022.878092)
Supplement: Supplementary file 1 [file Table1.docx]

Supplementary Table 1

ICD9/10 diagnosis codes that were identified by the Nova Scotia Dementia Strategy as most likely to identify an individual with a diagnosis of dementia

| **Description** | **ICD-9** | **ICD-10** |
| --- | --- | --- |
| Alcohol-induced persisting amnestic disorder | 290.x | F01.x, F05.1 |
| Alcohol-induced persisting dementia | 291.1 | F10.6 |
| Amnestic disorder in conditions classified elsewhere | 291.2 | F10.7 |
| Dementia in conditions classified elsewhere | 294.0 | F04.x |
| Other cerebral degenerations  ***Includes****: Alzheimer’s disease; Frontotemporal dementia; Senile degeneration of the brain; Communicating hydrocephalus; Idiopathic normal pressure hydrocephalus; Cerebral degeneration in diseases classified elsewhere; dementia with Lewy’s bodies; Dementia with Parkinsonism; Cerebral degeneration, unspecified.*  ***Excludes****: Obstructive hydrocephalus; Reye’s syndrome* | 331.0-331.3, 331.5-331.7, 331.82, 331.83, 331.89, 331.9 | G30.x, G31.0, G31.1, G31.8, G31.9, G32.8, G91.0, G91.2-G91.3, G91.8, G91.9, G94.x |
| Senility without mention of psychosis | 797 | R54.x |

*Adapted from the Nova Scotia Dementia Strategy*
